# Measuring windows of selection for anti-malarial drug treatments

**DOI:** 10.1186/s12936-015-0810-4

**Published:** 2015-07-31

**Authors:** Katherine Kay, Ian M Hastings

**Affiliations:** Parasitology Group, Liverpool School of Tropical Medicine, Pembroke Place, Liverpool, L3 5QA UK

**Keywords:** Malaria, Drug resistance, Window of selection, Pharmacokinetic, Pharmacodynamic, Modelling, Artemisinin combination therapy

## Abstract

**Background:**

The long half-lives of malaria ‘partner’ drugs are a potent force selecting for drug resistance. Clinical trials can quantify this effect by estimating a window of selection (WoS), defined as the amount of time post-treatment when drug levels are sufficiently high that resistant parasites can re-establish an infection while preventing drug-sensitive parasites from establishing viable infections.

**Methods:**

The ability of clinical data to accurately estimate the true WoS was investigated using standard pharmacokinetic–pharmacodynamic models for three widely used malaria drugs: artemether–lumefantrine (AR-LF), artesunate–mefloquine (AS-MQ) and dihydroartemisinin–piperaquine (DHA-PPQ). Estimates of the clinical WoS either (1) ignored all new infections occurring after the 63-day follow-up period, as is currently done in clinical trials, or, (2) recognized that all individuals would eventually be re-infected and arbitrarily assigned them a new infection day.

**Results:**

The results suggest current methods of estimating the clinical WoS underestimate the true WoS by as much as 9 days for AR-LF, 33 days for AS-MQ and 7 days for DHA-PPQ. The new method of estimating clinical WoS (i.e., retaining all individuals in the analysis) was significantly better at estimating the true WoS for AR-LF and AS-MQ.

**Conclusions:**

Previous studies, based on clinically observed WoS, have probably underestimated the ‘true’ WoS and hence the role of drugs with long half-lives in driving resistance. This has important policy implications: high levels of drug use are inevitable in mass drug administration programmes and intermittent preventative treatment programmes and the analysis herein suggests these policies will be far more potent drivers of resistance than previously thought.

**Electronic supplementary material:**

The online version of this article (doi:10.1186/s12936-015-0810-4) contains supplementary material, which is available to authorized users.

## Background

Artemisinin combination therapy (ACT) is effective, first-line treatment for uncomplicated *Plasmodium falciparum* malaria and now widely deployed in most endemic countries [1]. The artemisinin component is extremely fast acting and highly potent but rapidly eliminated so current ACT all contain a second ‘partner’ drug with a longer half-life. The long half-lives of the partner drugs are clinically beneficial: they may persist at active concentrations for weeks after treatment, providing valuable prophylaxis against infection during this period [2, 3]. However, the rapid elimination of artemisinin means the partner drugs are present as a monotherapy during this prophylactic period, which may be a potent force selecting for resistant parasites. This process can drive increasing drug tolerance to the partner drug and the eventual loss of therapeutic effectiveness, resulting in failure of the ACT [4–6]. This ability of parasites to evolve resistance to the partner drug is a possible Achilles heel of ACT.

The putative consequences of long half-life partner drugs driving resistance enters debates on the politics of anti-malarial drug deployment. Mass drug deployment (MDA) policies were widely used in the 1950s and 1960s (reviewed by Von Seidlein and Greenwood [7]), but fell from favour as, used in isolation, they had only a transitory effect on malaria transmission and drove drug resistance to high levels. MDA policies are once again under active consideration as part of a comprehensive toolkit for eradicating malaria populations [8], particularly those believed to have evolved artemisinin resistance [9–11]. Intermittent preventative treatment (IPT) programmes also deploy anti-malarial drugs and have proven highly effective in protecting vulnerable populations [12, 13], but as malaria transmission declines it is unclear at what point their short-term clinical benefit is outweighed by the putative longer-term consequences of driving resistance [13]. Anti-malarial drug use in MDA and IPT programmes drives resistance in two ways: (1) they select for parasites able to survive treatment; and, (2) their long half-lives mean they persist for long periods after treatment, selecting for newly acquired resistant parasites able to survive residual drug levels as they emerge from the liver and attempt to establish a viable infection. Mathematical modelling suggests the first force is easy to measure: it is simply, and intuitively, the proportion of existing asexual blood infections that are treated (often called simply the ‘drug coverage’). The second force is more difficult to quantify but note that the size of its effect is, somewhat counter-intuitively, not affected by local transmission intensity (Box 1 of [5]). The use of drugs in MDA and IPT is almost guaranteed to increase selection for resistance [7, 13, 14], but to what extent? This paper attempts to provide quantitative answers to this second force, selection due to residual drug levels, through the application of pharmacological modelling of anti-malarial drug treatment.

The genetic process whereby parasites evolve increasing tolerance to the partner drug is usually quantified as a window of selection (WoS). As a specific example, Watkins and Mosobo [6] noted parasites with the *dhfr*108 mutation could be observed in patients 15 days after treatment with sulfadoxine-pyrimethamine (SP), whereas wild-type infections were only observed after 50 days, thus implying a WoS of 35 days. Similarly, Sisowath et al. [3] estimated a WoS of 15 days associated with the *pfmdr1* D1246Y mutation after lumefantrine treatment. Routine genotyping in clinical trials means such data are readily available and have been used previously to estimate WoS [3, 4, 6, 15]. People in endemic areas often have residual drug levels resulting from previous chemoprophylaxis or direct treatment [16–18]. This is likely to result in frequent selection windows for most drugs. For example, if five courses of SP are taken per year and, as above, assuming a 35-day WoS, then there will be 5 × 35 = 175 days per person in which persisting drug concentrations are selecting for the *dhfr*108 mutation. This implies that selection for resistance via WoS may be widespread and intense.

**Fig. 1:**
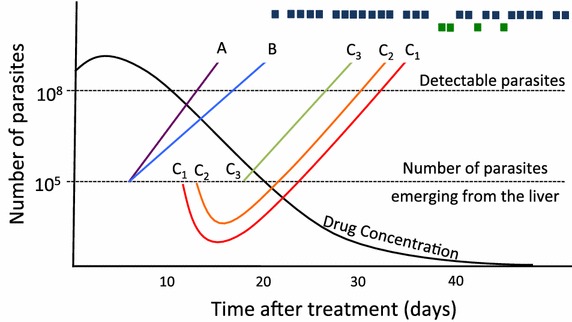
Potential problems with using patent parasitaemia to estimate ‘windows of selection’ (WoS). Field studies typically measure the WoS by comparing the times different genotypes become detectable (‘patent’) in patients. As an example, the clinical data of Sisowath et al. [3], showed that resistant parasites bearing the *pfmdr1* D1246Y mutation (*top row*; *blue squares*) first become patent in patients approximately 20 days after treatment and about 15 days earlier than sensitive parasites (*bottom row*; *green squares*). Each new infection was assumed to comprise 10^5^ parasites emerging from the liver and to become patent if/when their numbers reached 10^8^ parasites (*horizontal dashed lines*). The *black line* shows the decrease in drug concentration over time (using a single drug dose for illustration). *Lines A* and *B* show two clones that emerge on the same day but grow at different rates because of their differing IC50s and become patent several days apart. Here, field estimates based on patent parasitaemia would quantify a WoS of around 5 days when in fact there is none. *Lines C*_*1*_ *–C*_*3*_ illustrate how the earliest emerging parasites may not necessarily correspond to the first patent infection. *C*_*1*_ is the earliest emerging clone but residual drug levels cause an initial drop in parasite numbers and so *C*_*2*_, which emergences slightly later, is able to become patent sooner. Similarly, by the time *C*_*3*_ emergences from the liver, drug levels have fallen sufficiently that it no longer effects the newly emerged clone and so *C*_*3*_ becomes patent before both *C*_*1*_ and *C*_*2*_. The ‘clinical’ WoS is actually wrong: the true order of survival is *C*_*1*_, *C*_*2*_, *C*_*3*_ but the clinical order is *C*_*3*_, *C*_*2*_, *C*_*1*._

These WoS estimates, obtained from clinical observations, are widely cited and refered to here as ‘clinical’ WoS to denote their origin in clinical observations. In fact, the ‘true’ WoS is the period during which infections bearing the mutation can emerge from the liver (assuming the drugs do not kill the parasites while in the liver stage) and survive to produce a viable infection, while sensitive parasites are killed by residual drug concentrations [19]. Unfortunately, it is impossible to directly observe the ‘true’ WoS because the 10^5^ parasites that emerge from the liver are below patency. It has, therefore, been widely assumed that the clinical WoS reflects the true window (*op cit*). However, it is not clear how well the clinical WoS estimates the true WoS and there are several plausible reasons why they may be poor estimators, discussed further in Fig. 1. This paper uses the pharmacokinetic–pharmacodynamic (PK/PD) model described previously [20–22] to simulate the WoS for increasingly resistant infections, with the aim to quantifying how accurately clinical WoS estimate the ‘true’ window of selection.

## Methods

The mechanistic PK/PD model of Winter and Hastings [20] was used here with the additional absorption and conversion phases for the artemisinins, and the parameter-specific estimates of variation as described in Kay and Hastings [21]. The model was applied to three highly effective ACT regimens currently used to treat malaria: (1) artemether–lumefantrine (AR-LF), 1.7 mg/kg AR and 12 mg/kg LF given twice daily for 3 days; (2) artesunate–mefloquine (AS-MQ), 4 mg/kg AS and 8.3 mg/kg MQ given once daily for 3 days; and, (3) dihydroartemisinin–piperaquine (DHA-PPQ), 4 mg/kg DHA and 18 mg/kg PPQ given once daily for 3 days. Further details of the model methodology and parameterization are included in Additional file 1.

Mass drug treatment in human populations was simulated assuming 5,000 simulated patients were each given the same ACT; all patients were treated at day 0 and the simulations run for 365 days; 63 days is the recommended period of follow-up in clinical trials but the 365-day ‘follow-up’ in the simulations reveal how finishing patient observations at 63 days may affect the results. The focus was solely on increasing resistance to the partner drugs and this was incorporated into simulations by increasing the IC50 of parasites to the partner drug; hence ‘genotypes’ in this study refers to parasite genotypes with different values of IC50 to the partner drug. Any parasites present on the day of treatment (i.e., day 0) were ignored on the assumption that the ACT is clinically effective and would eliminate them.

**Fig. 2:**
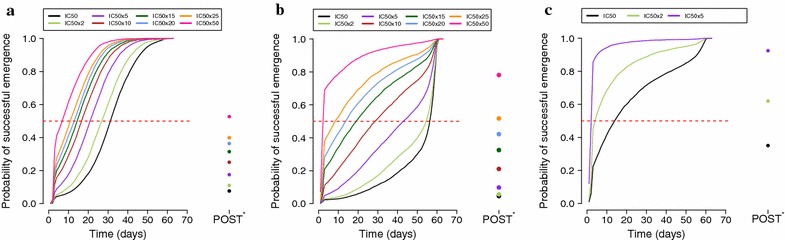
The true window of selection for three simulated anti-malarials drugs. The probability of parasites emerging from the liver and successfully surviving residual drug levels to cause a patent new infection is a function of (1) their drug resistance level, simulated as increasing levels of IC50 and, (2) the day they emerge after treatment. Three drug combinations are simulated: **a** artemether–lumefantrine, **b** artesunate–mefloquine and **c** DHA-piperaquine. Note the probabilities of surviving treatment (POST) with monotherapy are shown to immediate right of the x-axis.

The principle underlying the analyses is simple. First, the probability that parasites with different drug-resistant ‘genotypes’ emerging from the liver could survive residual drug levels on any given day post-treatment was established; this estimated the ‘true’ WoS (i.e., according to the calculations presented here, see discussion of Fig. 2). Then the time until infections of any given ‘genotype’ first became clinically detectable in the blood was determined; this estimated the ‘clinical’ WoS. This methodology can therefore be used to determine whether the clinically observed WoS accurately predicts the ‘true’ WoS. The true and clinical WoS was investigated as follows.

### True WoS

**Fig. 3:**
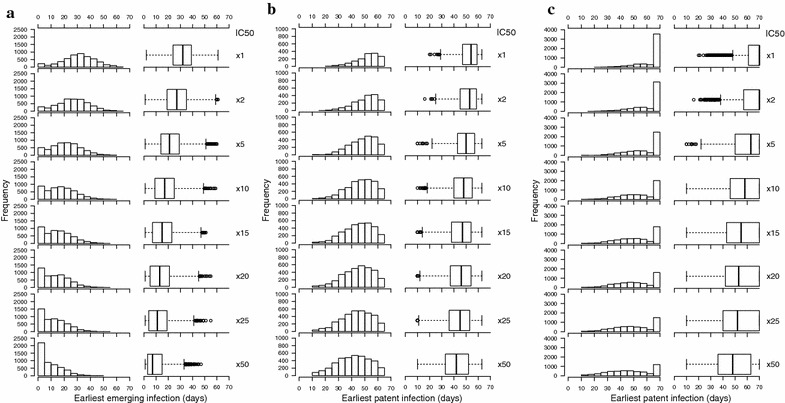
The window of selection (WoS) for artemether–lumefantrine (AR-LF). Data from 5,000 patients; the earliest successful emergence from the liver (which determines the ‘true’ WoS) is shown in **a**, the earliest observed patent infection (used to calculate the observed, ‘clinical’ WoS) determined using Method 1 is shown in **b** and using Method 2 is shown in **c**. The histogram and box plot in each *row* summarize the same data in different forms. Data plotted in the *top row* corresponds to the mean LF IC50 and data in subsequent *rows* shows the results of increasing the LF IC50. Patients were followed up for 365 days with the follow-up time censored when clinical WoS to 63 days, the maximum recommend time of a clinical trial. All new infections occurring after the 63-day follow-up period were either (1) removed from the analysis (**b**) or (2) re-classified arbitrarily as occurring at day 100 (**c**).

Each patient’s drug pharmacokinetic (PK) parameters were randomly assigned using the means and distributions in Additional file 1: Table S1 and each patient’s infection consisted of one parasite clone with the parasites pharmacodynamic (PD) parameters randomly allocated using the means and distributions given in Additional file 1: Table S1. Every day after treatment, 10^5^ parasites were assumed to emerge from the liver and encounter the residual drug levels persisting from the initial treatment; the fate of the clone emerging each day was recorded by noting whether or not it would eventually survive to form a viable infection, and therefore the earliest day on which successful emergence from the liver was first possible. This provided a distribution of earliest possible emergence days for the 5,000 human/parasite combinations (see later discussion of Fig. 2, and the left hand panels of Fig. 3 and Additional file 1: Figures S1, S2). This process was repeated to simulate ‘alleles’ encoding increasing drug resistance; each of the 5,000 patients retained their PK parameters to ensure consistency across alleles while the parasite PD parameters were re-assigned. Seven increasingly resistant alleles were simulated with the partner drugs IC50 increased 2, 5, 10, 15, 20, 25, and 50-fold greater than the mean (Additional file 1: Table S1); each allele therefore generated a distribution of 5,000 earliest emergence days.

The question then arises as to how to translate these distributions of the earliest emerging infections into a strict definition of the true WoS. It would be inappropriate to define the start of the WoS as the earliest possible emergence day in the distribution, because this will depend on chance and on sample size. Therefore the true WoS was defined as the difference between the day post-treatment on which 50% of clones could survive residual drugs for any IC50 ‘genotype’, i.e., the median of the distribution (see discussion of Fig. 2).

### Clinical WoS

Estimates of the clinical WoS were determined by assuming patients were followed for 63 days which is the longest follow-up period currently recommended in a clinical trial [23]. The 5,000 humans used above to simulate the true WoS retained their original PK parameters but the parasite PD parameters were re-assigned for each allele with increasing IC50 values. It was assumed that, on average, each patient would acquire 16 new infections per year; this was based on re-infection data from northern Ghana [24] (see Additional file 1 and later discussion for more details). For each individual, the number of new infections was chosen from a Poisson distribution with a mean value of 16. To maintain the population size, if an individual had no new infections (i.e., if a value of zero was chosen from the Poisson distribution) another number was selected in the same way. The day on which each of the new infections emerged from the liver during the follow-up period was randomly assigned. Each newly emerging infection had PD values chosen independently from Additional file 1: Table S1 so that, in patients with more than one new emergence, each emergence was genetically distinct (i.e., had different PD values around the assigned mean IC50). The fate of each emergence in a human was determined as before (survival/death and, if survival, time to clinical patency). The simulated patient was then categorized as having ‘no re-infection’ (if no patent infection appeared in the 63 days after treatment) or ‘re-infected’ with a time of first patency.

The method currently used to estimate clinical WoS is poorly defined and generally involves visual inspection of the data; for example, the data of Sisowath et al. [3] (included here on Fig. 1) show that ‘resistant’ parasites first become apparent around day 20 while sensitive parasites become apparent around day 35, implying a clinical WoS of 15 days. The problem with this method is that it depends largely on chance and the sample size of each allele (determined by its frequency). For example, if a single sensitive infection had been noted on day 25 then the estimated WoS would have fallen to 5 days. The methodology is formalized herein by estimating the clinical WoS using two methods.

Method 1 reflects the current approach of estimating the clinical WoS and considers only infections occurring during trial follow-up. Rather than visual inspection of the first day of observed patency, the clinical WoS is more formally defined by comparing the median (or other centile scores) of the patency data to reduce the element of chance. For example, the data of Sisowath et al. [3] on Fig. 1 would produce a WoS of approximately 5 days based on the median values of the emergence times (day 35 for resistant and 40 for sensitive).

Method 2 is analogous to the first and compares centile scores of each allele to estimate the clinical WoS. The one difference is that Method 2 recognizes that each patient would eventually have become re-infected with the allele if followed up for a sufficiently long time and hence that the re-infection data are censored. Patients with no re-infections in the 63-day follow-up period (i.e., the censored patients) were arbitrarily scored as having re-infections occurring on day 70. The comparison to obtain WoS relies on centiles (not averages) so it is immaterial which day after 63 is arbitrarily chosen provided the number of censored individuals is not above 50% (see discussion of ‘BF’ below and in Table 1).

**Table 1:** Estimates of the true and clinical window of selection

Drug	IC50	True WoS	Clinical WoS					
			Method 1, current			Method 2, new		
			10th	25th	50th	10th	25th	50th
AR-LF	Default to 15-fold	17	9	9	8	16	16	BF
	15-fold to 50-fold	8	6	6	5	7	8	8
AS-MQ	Default to 15-fold	37	5	4	6	BF	BF	BF
	15-fold to 50-fold	17	9	10	8	14	18	BF
DHA-PPQ	Default to twofold	10	3	3	3	5	9	BF
	Twofold to fivefold	1	5	5	7	7	10	14

Simulations of 5,000 patients, followed for 63 days after treatment, infected with increasingly drug-resistant parasites and treated with artemether–lumefantrine (AR-LF), artesunate–mefloquine (AS-MQ) or dihydroartemisinin–piperaquine (DHA-PPQ). True windows of selection (WoS) were calculated by comparing the days on which 50% of emergences were successful (red dashed lines on Fig. 2; full details in main text). Two methods were used to calculate the clinical WoS: All new infections occurring after the 63-day follow-up period were either removed from the analysis (Method 1) or re-classified arbitrarily as occurring at day 70 (Method 2). The clinical WoS for each method were reported using either the tenth, 25th or 50th centile cut-offs. The cumulative differences in WoS were calculated for each treatment in absolute units i.e. the duration (in days) of the WoS.

*BF* beyond follow-up.

Both methods therefore generated a distribution of times to first patency analogous to the data of Sisowath et al. [3] (Fig. 1). The tenth, 25th and 50th centile values of the distributions were obtained for each of the resistance alleles.

The probability of surviving direct treatment (POST) with the partner drug monotherapy was also recorded for the 5,000 patients to provide a baseline probability of successful treatment, against which the survival probabilities of later emerging parasites could be compared. It was assumed that between 10^10^ and 10^12^ parasites (chosen from a uniform distribution) from a single clone were present at the time of treatment, the outcome (i.e., survival/death) was simulated and the probability of each IC50 ‘genotype’ surviving direct treatment determined.

## Results

The ‘true’ WoS are shown on Fig. 2 and the raw data are given on the left hand panels of Fig. 3 and Additional file 1: Figures S1 and S2. All ACT regimens routinely cleared infections emerging from the liver on the first day post-treatment irrespective of their IC50 value (Fig. 2). Thereafter the probability of an infection successfully establishing in patients was highly dependent on the parasite IC50. The probability of surviving showed the largest increase between days 2 and 3. This is likely to be due to the presence of the artemisinin component on day 2 and its absence by day 3 (by convention, treatment starts on day 0 so artemisinins are present on days 0, 1 and 2). The probabilities that an established, patent infection survived direct treatment with the partner drug monotherapy are given in Fig. 2 for each resistant ‘genotype’ in the column labelled ‘POST’. The POST was always greater than the probability of parasites surviving emergence on day 0 for two plausible reasons. Firstly, the established, patent infections treated on day 0 are present in much greater numbers (10^10^–10^12^ parasites) than the newly emerging infections on day 0 (10^5^ parasites). Secondly, POST is calculated for the partner drug monotherapy, whereas emergence on day 0 encounters both partner drug and an artemisinin derivative.

The presence or absence of a true WoS between the different genotypes can be determined from Fig. 2. As expected, the true WoS is closed in the initial days post-treatment (survival probability is around zero for all alleles) and closed again at later time points (when all alleles have a near 100% probability of surviving). The WoS is however open during the intermediary days and, interestingly, the probability changes between genotypes are roughly parallel. The true WoS was therefore arbitrarily defined as the time difference between the days when an allele has a 50% change of successful emergence (the dashed lines in Fig. 2).

The pattern of treatment outcome following AR-LF (Fig. 2a) was broadly similar to that noted with AS-MQ (Fig. 2b). Both ACT regimens kept the probability of successful emergence below 10% with sensitive infections (i.e., IC50 increases <fivefold) for ten to 20 days post-treatment (AR-LF and AS-MQ, respectively). As expected, the probability of successful emergence increased as infections became more resistant. The probability of an infection successfully becoming established just 10 days after treatment with the most resistant genotypes (50-fold IC50 increase) was as high as 60–80% following AR-LF and AS-MQ treatment, respectively. Treatment with DHA-PPQ was much more sensitive to increases in IC50 (Fig. 2c) than AR-LF and AS-MQ, suggesting it had a much shorter period of post-treatment prophylaxis (discussed later). Ten days after treatment, infections with PPQ-sensitive parasites had a 40% chance of successful emergence while infections with an IC50 increase of twofold and fivefold had a 68 and 95% chance of resulting in a successful emergence. Twenty days post-treatment, these probabilities increased to 60, 80 and 97%, respectively. Given that the probability of acquiring a new infection associated with a fivefold IC50 increase reached >95% almost immediately after the artemisinin component (DHA) was eliminated, it was unnecessary to illustrate greater increases in PPQ IC50 as the results would follow the same pattern.

The clinical WoS were formally defined as the differences between genotypes based on the tenth, 25th and 50th (i.e., median) centile values of patient data and are given on Additional file 1: Table S2 with the raw data shown on the middle and right hand panels of Fig. 3 for AR-LF, and Additional file 1: Figures S1 and S2 for AS-MQ and DHA-PPQ, respectively. Figure 3 shows the clinical WoS for AR-LF opens much later using Method 2 (right hand panel) than Method 1 (central panel) and shifts the distribution of earliest patent infections (used to define the opening of the WoS) significantly to the right (i.e., to later days). Additional file 1: Figures S1 and S2 show this result was consistent following AS-MQ and DHA-PPQ treatment.

The key research question addressed here is whether the clinical WoS reliably estimates the ‘true’ WoS; the estimates are given on Table 1 and Additional file 1: Tables S2 and S3. This does not appear to be true if the clinical WoS is estimated using Method 1, which ignored new infections occurring after the follow-up period (Table 1 and Additional file 1: Tables S2, S3). Following treatment, the clinical WoS consistently underestimated the true WoS by as much as 9 days for AR-LF, 33 days for AS-MQ and 7 days for DHA-PPQ (Method 1, Table 1). One notable exception to this rule occurs when DHA-PPQ resistance is increased from twofold to fivefold, the clinical WoS over estimates the true WoS by up to 6 days (Method 1, Table 1).

Calculating the clinical WoS using Method 2 generally provided better estimates of the true WoS (Table 1 and Additional file 1: Tables S2, S3). However, alleles that were particularly sensitive to the drug had such a low probability of becoming patent during the 63-day follow-up that the earliest patent infection (for a given centile value) often fell within the group of censored data arbitrarily assigned to day 70; the WoS was recorded as occurring ‘beyond follow-up’ (BF) in these circumstances. Following treatment with AR-LF, the clinical WoS accurately estimated the true WoS for all IC50 increases when defined using the tenth or 25th centile and for larger LF IC50 increases (i.e., >15-fold) defined using the 50th centile (Method 2 in Table 1 and Additional file 1: Table S2). If MQ IC50 was increased twofold or more, the tenth and 25th centile estimates of WoS were accurate to within one to 2 days (depending on the magnitude of the IC50 increase and the centile cut-off, Method 2, Additional file 1: Table S2). When MQ IC50 was equal to the default value, or when the 50th centile cut-off was used to define WoS, estimates of the AS-MQ clinical WoS were not possible as new infections occurred beyond the follow-up period (Method 2 in Table 1 and Additional file 1: Table S2). Estimates of clinical WoS following DHA-PPQ treatment were accurate when PPQ IC50 increased twofold (defined by the 25th centile, Method 2, Table 1) but overestimate the true WoS by as much as 6–13 days when IC50 increases from two to fivefold.

In summary, Table 1 shows that clinical WoS calculated using Method 1 (i.e., as currently estimated) were consistently shorter than the true WoS. Clinical WoS calculated using Method 2 were far more consistent with the true WoS; it appears that Method 2 based on the 25th centile provides the best match between true and clinical WoS. The exception was DHA-PPQ, which was overestimated by both methods when PPQ IC50 was increased more than twofold.

These results were determined assuming patients acquire 16 new infections per year (see Additional file 1 for details); note results were consistent when the number of new infections was reduced to eight per year (Additional file 1: Table S3).

## Discussion

The aim of this paper was to investigate how accurately genotyping data obtained during anti-malarial drug clinical trials can estimate WoS. The results show that the current method of estimating WoS from the clinically observed patency of different genotypes is a poor surrogate and tends to underestimate the true WoS (Method 1, Table 1). For example, the duration of the true WoS following a default to 15-fold IC50 increase was 17 and 37 days for AR-LF and AS-MQ, respectively; the corresponding clinical WoS estimates were eight to 9 days or 4–6 days, respectively (Method 1, Table 1). Including ‘censored’ individuals in the analysis (Method 2, Table 1) greatly improved clinical WoS estimates, particularly when using the 25th centile cut-off. Practically, Method 2 requires a significant number (ideally 10–25% of patients) of new infections with sensitive parasites to occur during the 63-day follow-up period to prevent estimates falling into the censored BF period which would preclude estimating WoS. For example, it was impossible to determine a WoS for MQ-sensitive parasites as new infections rarely occurred during the follow-up period; Fig. 2 shows there is still only a 30% chance of acquiring a new infection with MQ-sensitive parasites 50 days after the initial treatment.

A large discrepancy did occur for PPQ when IC50 was increased from two to fivefold; the clinical WoS estimates of the true WoS were greatly overestimated regardless of the method or centile used. Lines A and B on Fig. 1 provide one possible explanation for this. The true WoS (Table 1) shows infections with a PPQ IC50 increased twofold can emerge 1 day after infections with a fivefold IC50 increase. If these infections grow at different rates as a result of their differing IC50s then the infections would become patent several days apart and so the clinical WoS would be expected to overestimate the ‘true’ WoS.

The choice of model parameters was previously validated for these ACT [20–22] and the results generated herein are again highly consistent with field data. Fully effective ACT was able to clear infections with either sensitive or mildly resistant parasites when treated directly. Subsequent re-infections with LF-sensitive parasites typically become patent 25–35 days post-treatment in these simulations (central panel, Fig. 3), which closely match those reported by Sisowath et al. [2, 3] who found re-infections with LF-sensitive parasites occurring 24–30 days after treatment. The simulated results also show AS-MQ was able to prevent new infections by sensitive and mildly resistant parasites for longer than AR-LF. This implies MQ has the longer post-treatment prophylactic period, consistent with the data of Sagara et al. [25] who reported re-infections in Peru occur less frequently in their follow-up period of 28 days following AS-MQ treatment than AR-LF.

A previous study [26] used a similar PK/PD approach to elucidate the general principles underlying antimalarial drug windows of selection. This manuscript extends this seminal work in three important ways. Firstly, by incorporating recent methodological advances in PK/PD modelling of antimalarial dosing: this allows multiple dosing, drug absorption and conversion processes, and the co-administration of artemisinin as in the current generation of ACTs [20, 21]. It also allows the analysis of PPQ, a currently widely used antimalarial, which has more complicated (two or three compartmental) PK (e.g. [27]). It is unlikely that these inclusions will have a large qualitative impact on the basic properties of WoS but it is important to repeat the analyses using the current best practice in PK/PD modelling. Interestingly, there were some notable quantitative differences: unlike the Stepniewska and White paper [26], the new analyses did not find WoS that lasted “several months”. The estimates of WoS presented here last approximately 1–2 weeks; this has important policy implications as shorter WoS suggest a slower spread of drug resistance. The second advance is the simultaneous inclusion of the substantial variance that occurs in all the PK/PD parameters (Additional file 1: Table S1); this reflects the reality in vivo and means that WoS become probabilistic (Fig. 2) rather an fixed entity, and requires that WoS be re-defined in this more probabilistic context. Finally, simulations included re-infection of patients to show how WoS may be estimated from clinical data. It is now considered mandatory in malaria drug clinical trials to genotype recurrent malaria to distinguish treatment failures from new infections [28]. Recent advances in identifying candidate genes encoding resistance [29, 30] mean that these samples can later be re-genotyped at candidate genes to obtain clinical evidence of their impact on resistance. The new method of analysis (Method 2) will allow these data to be analyzed in the appropriate, accurate manner.

One objective of this study was to contribute to the theory describing the dynamics underlying the spread of anti-malarial drug resistance. Three processes drive drug resistance: the ability to survive treatment, the WoS and intra-host dynamics [31]. The ability of a mutation to survive treatment can already be quantified by clinical trials. Quantifying intra-host dynamics is less easy although some progress has been made in mouse models [32] and through inference from clinical data [33]. The results presented here on WoS suggest that it is now possible to accurately quantify all three of the forces driving resistance. Table 1 suggests that the current method of estimating clinical WoS by ‘visual inspection’ or its more formalized Method 1 described above, underestimate the ‘true’ WoS for AR-LF, AS-MQ. The new Method 2 includes all patients when estimating the clinical WoS and obtains estimates much more consistent with the ‘true’ WoS. It is however important to consider that methodologically, Method 2 requires that estimates of WoS be undertaken in areas of high transmission to ensure a reasonable proportion of the population acquire a new infection during the follow-up period (see recommendations below). Quantifying this impact for the current generation of anti-malarials can only be achieved through accurate estimation of their true WoS, hence the importance of verifying whether clinical WoS accurately reflect the true WoS.

The results presented here are only applicable to the three drug regimens investigated. Researchers are urged to use the methodology contained within this and previous papers [20–22] to estimate the likely clinical WoS for other drugs and/or drug regimens. By reproducing the plots in Fig. 2 for their chosen drug regimens, researchers can quickly and cheaply assess the likely success of estimating WoS from field data. For example, it would not be advisable to estimate the WoS for drugs where the true WoS is likely to be very short (such as PPQ; Fig. 2c) but it would be suitable to use the new method, Method 2, for drugs that have longer true WoS (Fig. 2a, b, respectively). This new methodology does also require that clinical trials be carried out in areas of moderate to high transmission in which both, or all, alleles are well represented so patients have an adequate chance of acquiring a new infection.

The study also has operational implications for anti-malarial drug deployment policies. The long half-lives characteristic of most malaria partner drugs have always been regarded as potent drivers of drug resistance but the results presented here suggest they are more potent drivers than implied by previous estimates of WoS based on ‘visual-inspection’ of re-infections (or the more formal Method 1). There is currently interest in the improved diagnostics of malaria treatment, primarily to ensure treatment is restricted to confirmed malaria cases, so treatment is appropriate to the underlying clinical condition [8]. A secondary objective is to reduce the use of expensive ACT and, it is argued, reduce “selection pressure for drug resistance” [34]. For example, if a drug had a WoS of 15 days and a patient was given six treatments throughout the year, there would be 15 × 6 = 90 days or approximately 25% of the year in which the resistant parasites were being preferentially selected; cutting treatment down to two doses per year would obviously decrease this selection by two-thirds, i.e., to 15 × 2 = 30 days. The results suggest the decreased selection for resistance caused by improved diagnostics may be much greater than anticipated. This is because the drugs’ true WoS are longer and potentially much more important drivers of resistance than suggested by analysis of clinical data using Method 1. The results also suggest that proposed drug deployment policies based on mass distribution of ACT, such as MDA and IPTs, which inevitably increases drug use in the population, have the potential to be more potent drivers of resistance than has been previously thought because the WoS are longer than anticipated. A detailed investigation of the advantages and disadvantages of such interventions is outside the remit of this paper, but it does emphasise that accurately quantifying the magnitude of WoS has both a broad application in quantifying the drivers of drug resistance, and also has a more specific role in the evaluating the likely impacts of interventions such as IPT and MDA.

## Conclusion

This study was designed to identify the best methods to analyse clinical data that generate results consistent with the ‘true’ WoS. The resulting recommendations are as follows. First, if possible, a PK/PD analysis similar to that shown in Fig. 2 should be performed to obtain estimates of the true WoS for the chosen drug treatment. Extreme caution would be required when interpreting WoS estimates obtained for treatments that are particularly sensitive to increasing drug resistance (such as PPQ in these examples). Second, the clinical data should be analysed using Method 2 described above; this includes censored patients who do not have a new infection during follow-up. However, the potential problem with Method 2 is that the classification centile may lie in the BF class (Table 1). Hence accurate WoS estimates are only really feasible if trials are carried out in areas of moderate to high transmission where censored patients are less than 75% of the population, i.e., at least 25% of patients get re-infections during the follow-up. Thirdly, the analyses should be done in areas where both, or all, alleles are present at significant frequencies to allow a direct comparison in the same clinical setting.

## Additional file

Additional file 1: Includes data that support and expand some of the interpretations and conclusions drawn in the main text, but whose inclusion would detract from the main argument.

## Abbreviations

ACT: artemisinin combination therapy
AR: artemether
AS: artesunate
DHA: dihydroartemisinin
*dhfr*: dihydrofolate reductase
IC50: concentration required to produce half the desired effect
IPT: intermittent preventative treatment
LF: lumefantrine
MDA: mass drug administration
MQ: mefloquine
nM: nanomolar
PD: pharmacodynamic
*Pfcrt*: *Plasmodium**falciparum* chloroquine resistant transporter
*Pfmdr1*: *Plasmodium**falciparum* multidrug resistant protein-1
PK: pharmacokinetic
POST: probability of surviving treatment
PPQ: piperaquine
RDT: rapid diagnostic test
SP: sulfadoxine-pyrimethamine
WoS: window of selection
