# Supplementary material for: Measuring windows of selection for anti-malarial drug treatments
Source: Malar J. 2015 Jul 31;14:292. doi: 10.1186/s12936-015-0810-4 (PMC4521485; doi:10.1186/s12936-015-0810-4)
Supplement: Additional file 1: — Includes data that support and expand some of the interpretations and conclusions drawn in the main text, but whose inclusion would detract from the main argument. [file 12936_2015_810_MOESM1_ESM.pdf]

Additional file 1

This Additional file includes data that support and expand some of the interpretations and conclusions drawn in the main text, but whose inclusion would detract from the main argument.

The modelling methodology is described in more detail in previous publications [1-3]. Implemented in R [4], the model tracks parasite number as a function of parasite growth and changing drug concentration. Simulations are run in half-day time steps for the first seven days to allow for multiple dosing and one day time steps thereafter to reduce simulation time. The required pharmacokinetic (PK) and pharmacodynamic (PD) parameters were originally described in Winter & Hastings [1] assuming that all the drugs were instantaneously absorbed, not converted to an active form and have one structural PK compartment. The parameter specific estimates of variation and the additional parameters required to allow for AR or AS absorption and conversion were described in Table A1 of Kay & Hastings [2]. The mean volume of distribution for AR and DHA was subsequently updated by Hodel *et al.* [3] to improve the simulations estimates of cure rates for AR monotherapy (for more details see [3]). The PK structure of PPQ is better described by a two- or three-compartment model, here we used a two-compartment PK model described by Hodel *et al*. [5] and validated in [6]. Resistance to the artemisinins was not considered because they are present in the human body for such a short time that they are unlikely to affect the WoS for their partner drugs; however the artemisinin component was included in simulations to determining the fate of parasites emerging from the livers on days 1, 2 and 3.

When simulating clinical trials we used data from Northern Ghana to estimate the average number of new infections an individual acquires each year [8] which was reported to be 16. Other estimates can be crudely obtained using data on effective ACTs. For example, Burkirwa *et al.* (3) report more than 50% of patients developed recurrent parasitaemia within a month of treatment. Assuming reinfections can occur at any time in the month following treatment, it is predicted that each patient will acquire approximately 6 new infections each year. However, patients were known to be taking effective ACTs with long half lives and so it can crudely be assumed no new infections became established for the first two weeks following treatment (due to their long half-life). It can then be assumed 50% of patients acquire new infections in a two week period and so are more likely to receive an average of twelve new infections each year. More recently, Mueller *et al.* [9] have measured reinfection rates in an area of moderate transmission in Papua New Guinea and show individuals acquire approximately six new infections per year. The results shown here are consistent for patients who acquire either 16 (Table 1, main text) or 8 (Table A3) new infections per year. Note, lower re-infection rates were not simulated because clinical trials run in areas of low transmission will have so few new infections during follow up that it would not be possible to estimate a clinical WoS.

Note that when simulating field data (to determine the 'clinical' WoS) heterogeneity in the biting rate was ignored and hence (presumably) the heterogeneity in the rate at which people become re-infected in the simulated clinical trials. Note also that this is the number of <u>successful</u> re-infections per year. The number of potentially infectious bites per year (normally quantified by the entomological inoculation rate, EIR) may be much higher often ranging between 100 and 500. The discrepancy presumably

arises because most infectious bites do not result in establishing an infection, presumably because of acquired immunity in these high transmission areas.

References

1. Winter K, Hastings IM: **Development, evaluation, and application of an *in silico* model for antimalarial drug treatment and failure.** *Antimicrob Agents Chemother* 2011, **55:**3380-3392.

2. Kay K, Hastings IM: **Improving pharmacokinetic-pharmacodynamic modeling to investigate anti-infective chemotherapy with application to the current generation of antimalarial drugs.** *PLoS Comput Biol* 2013, **9:**e1003151.

3. Hodel EM, Kay K, Hayes D, Terlouw D, Hastings I: **Optimizing the programmatic deployment of the anti-malarials artemether-lumefantrine and dihydroartemisinin-piperaquine using pharmacological modelling.** *Malar J* 2014, **13:**138.

4. **R: A language and environment for statistical computing** [http://www.R-project.org/]

5. Hodel EMS, Guidi M, Zanolari B, Mercier T, Duong S, Kabanywanyi A, Ariey F, Buclin T, Beck H-P, Decosterd L, et al: **Population pharmacokinetics of mefloquine, piperaquine and artemether-lumefantrine in Cambodian and Tanzanian malaria patients.** *Malar J* 2013, **12**.

6. Kay K, Hodel EM, Hastings IM: **Altering drug regimens to restore and enhance antimalarial drug effectiveness.** 2015 (submitted).

7. Sisowath C, Ferreira PE, Bustamante LY, Dahlström S, Mårtensson A, Björkman A, Krishna S, Gil JP: **The role of *pfmdr1* in *Plasmodium***

***falciparum* tolerance to artemether-lumefantrine in Africa.** *Tropical Medicine & International Health* 2007, **12:**736-742.

8. Sama W, Owusu-Agyei S, Felger I, Vounatsou P, Smith T: **An immigration–death model to estimate the duration of malaria infection when detectability of the parasite is imperfect.** *Statistics in Medicine* 2005, **24:**3269-3288.

9. Mueller I, Schoepflin S, Smith TA, Benton KL, Bretscher MT, Lin E, Kiniboro B, Zimmerman PA, Speed TP, Siba P, Felger I: **Force of infection is key to understanding the epidemiology of *Plasmodium falciparum* malaria in Papua New Guinean children.** *Proc Natl Acad Sci* 2012, **109:**10030-10035.

Table A1. Mean antimalarial drug pharmacokinetic and pharmacodynamic parameters. Mean patient drug parameters and associated co-efficient of variation (in square brackets) for artesunate, artemether, DHA, lumefantrine, mefloquine and piperaquine.

	Artemether – Lumefantrine			Artesunate – Mefloquine			DHA - Piperaquine	
	AR	DHA	LF	AS	DHA	MQ	DHA	PPQ
Vd_c	46.6 [0.82]	15.0 [0.48]	21.0 [2.63]	7.1 [0.94]	1.49 [48]	20.8 [0.38]	1.49 [48]	346.0 [0.93]
Vd_p	-	-	-	-	-	-	-	443.0 [1.70]
k_a	23.98 [0.68]	-	-	252 [1.12]	-	-	-	11.2 [2.17]
k_m	11.98 [0.65]	-	-	30.96 [0.36]	-	-	-	-
k_{el}	-	44.15 [0.23]	0.16 [0.05]	-	25.4 [0.23]	0.053 [0.63]	19.8 [0.23]	-
CL	-	-	-	-	-	-	-	$108 \cdot BW^{0.75}$ [1.01]
Q_1	-	-	-	-	-	-	-	69.7 [1.01]
IC_{50}	0.0023 [0.79]	0.009 [117]	0.032 [102]		0.009 [117]	0.027 [0.78]	0.009 [117]	0.02 [0.3]
V_{max}	27.6 [0.3]	27.6 [0.3]	3.45 [0.3]	27.6 [0.3]	27.6 [0.3]	3.45 [0.3]	27.6 [0.3]	3.45 [0.3]
n	4.0 [0.3]	4.0 [0.3]	4.0 [0.3]	4.0 [0.3]	4.0 [0.3]	5.0 [0.3]	4.0 [0.3]	6.0 [0.3]

Vd_c: central volume of distribution [L/kg]; Vd_{p1}: volume of distribution [L/kg] in peripheral compartment 1; k_a: absorption rate per day; k_m: conversion rate per day of parent drug to metabolite; k_{el}: elimination rate per day ($k = CL/Vd_c$); CL: clearance [L/day]; Q_1: inter-compartmental clearance [L/day/kg] between the central and peripheral compartment 1; IC_{50}: concentration required to produce half the desired effect [mg/L]; V_{max}: first-order rate constant of parasite killing [/day]; n: slope of the concentration-effect curve; BW: body weight [kg] of the typical patient in the original study.

Table A2. Estimates of the true and clinical window of selection. Simulations of 5000 patients, followed for 63 days after treatment, infected with increasingly drug resistant parasites and treated with artemether-lumefantrine (AR-LF), artesunate-mefloquine (AS-MQ) or dihydroartemisinin-piperaquine (DHA-PPQ).Clinical windows of selection (WoS) were calculated using either the 10th, 25th, or 50th centile of their WoS distributions; full details in main text.

Drug	IC50	True WoS	Clinical WoS					
			Method 1			Method 2		
			10th	25th	50th	10th	25th	50th
AR-LF	default to 2-fold	5	1	1	2	4	4	BF
	2-fold to 5-fold	6	2	3	2	5	5	BF
	5-fold to 10-fold	4	4	3	3	4	5	BF
	10-fold to 15-fold	2	2	2	1	3	2	2
	15-fold to 20-fold	2	0	1	2	1	2	2
	20-fold to 25-fold	2	2	2	0	2	2	2
	25-fold to 50-fold	4	4	3	3	4	4	4
AS-MQ	default to 2-fold	2	1	-1	1	BF	BF	BF
	2-fold to 5-fold	12	-1	1	1	11	BF	BF
	5-fold to 10-fold	14	2	2	1	7	BF	BF
	10-fold to 15-fold	9	3	2	3	6	7	BF
	15-fold to 20-fold	6	1	2	1	3	4	BF
	20-fold to 25-fold	5	2	2	2	3	4	BF
	25-fold to 50-fold	6	6	6	5	8	10	13

Table A3. Estimates of the true and clinical window of selection. As in Table 1 of the main text except patients were assumed to acquire approximately eight new infections per year. This allowed us to identify whether reduced infection rates had any effect on the 'true' WoS estimates

Drug	IC50	True WoS	Clinical WoS					
			Method 1			Method 2		
			10th	25th	50th	10th	25th	50th
AR-LF	default to 15-fold	17	9	8	5	17	BF	BF
	15-fold to 50-fold	8	5	6	5	7	8	BF
AS-MQ	default to 15-fold	37	6	5	4	BF	BF	BF
	15-fold to 50-fold	17	9	9	7	16	BF	BF
DHA-PPQ	default to 2-fold	10	3	3	4	8	11	BF
	2-fold to 5-fold	2	5	5	5	7	12	BF

BF = beyond follow-up

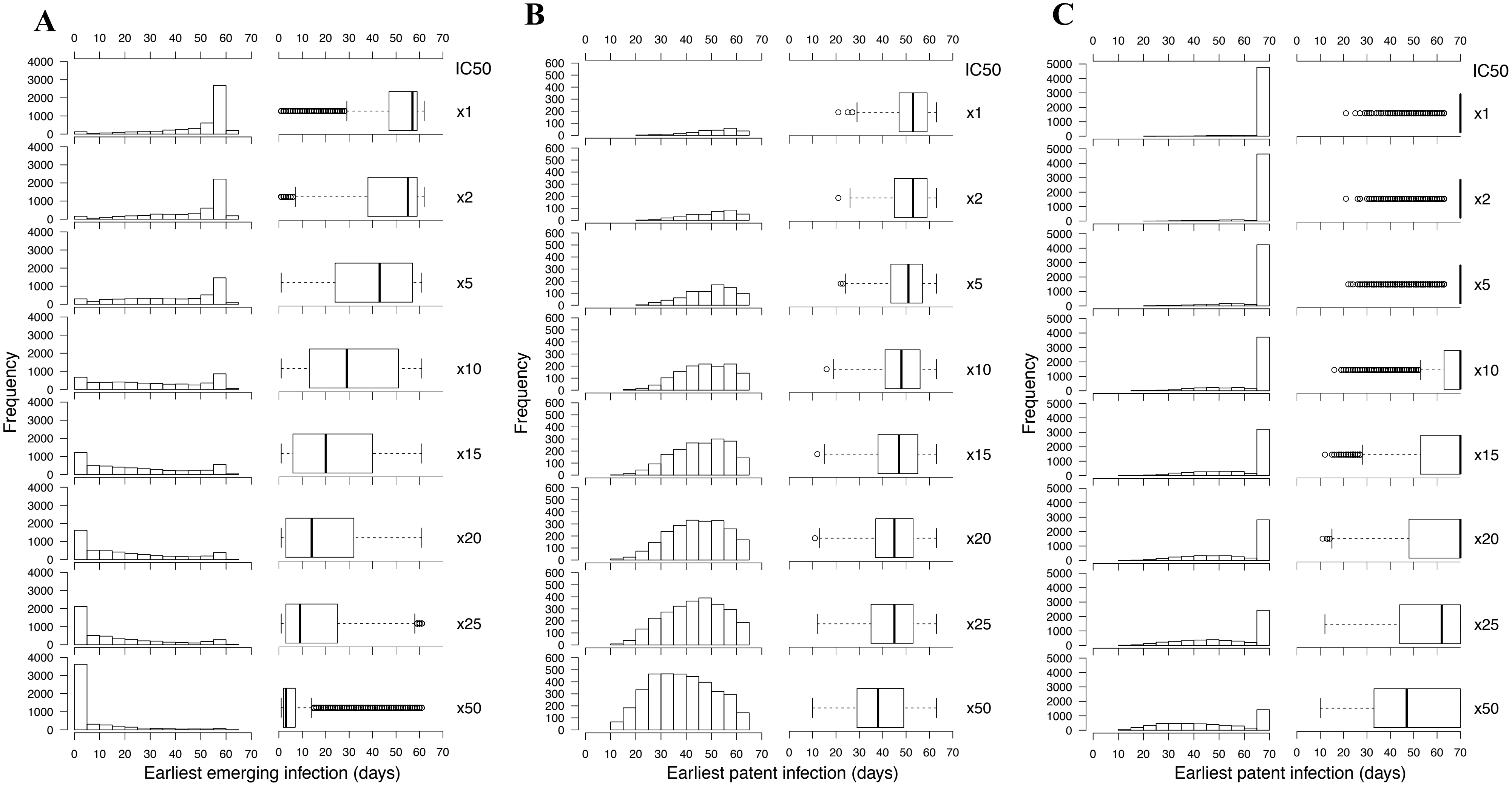

Figure A1. The window of selection for artesunate-mefloquine. See Figure 3 of the main text for more details.

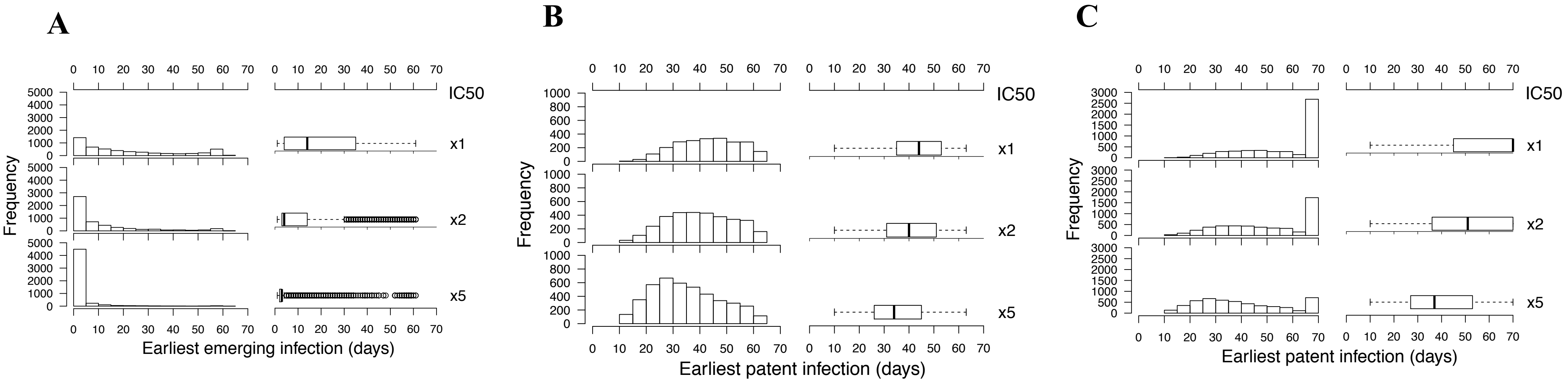

Figure A2. The window of selection for DHA-piperaquine. See Figure 3 of the main text for more details.
